# In Vitro Activity and Synergism of Amphotericin B and the Synthetic Bioinspired Peptide _γ31-45_*Pv*D_1_^++^ Against Fungal Study Models *Candida tropicalis* and *Saccharomyces cerevisiae*

**DOI:** 10.1007/s00284-026-05197-2

**Published:** 2026-10-21

**Authors:** Thaynã A. M. Souza, Érica de O. Mello, Fernanda G. Trindade, Felipe F. Moreira, Gabriel B. Taveira, Milena B. Cherene, Larissa M. Resende, Sérgio H. Seabra, Maura da Cunha, André de O. Carvalho, Valdirene M. Gomes

**Affiliations:** 1 Laboratório de Fisiologia e Bioquímica de Microrganismos, Centro de Biociências e Biotecnologia, Universidade Estadual do Norte Fluminense Darcy Ribeiro, Campos dos Goytacazes, RJ 28013-602 Brazil; 2 Laboratório de Biologia Celular e Tecidual, Centro de Biociências e Biotecnologia, Universidade Estadual do Norte Fluminense Darcy Ribeiro, Campos dos Goytacazes, RJ 28013-602 Brazil

## Abstract

In recent years, following the COVID-19 pandemic, the emergence of antibiotic-resistant microorganisms has increased. This surge has prompted global research into new biologically active molecules, particularly those focused on combination therapies involving antifungal agents. Synthetic antimicrobial peptides have emerged as promising candidates to address this demand. Therefore, the objective of this study was to assess the synergistic therapeutic effects of the synthetic peptide γ_31-45_*Pv*D_1_^++^ combined with amphotericin B (AmB) on *Candida tropicalis* and *Saccharomyces cerevisiae*. This study aimed to identify the synergistic concentration that could facilitate advancement in the development of novel drugs. Here, we demonstrated that synergistic combinations of synthetic peptides and amphotericin B (AmB) effectively induce cell death in *C. tropicalis* and *S. cerevisiae*. The checkerboard test revealed synergistic concentrations of 0.048 µM for AmB, 0.39 µM for the synthetic peptide γ_31-45_*Pv*D_1_^++^ for *C. tropicalis*, 0.097 µM for AmB, and 0.78 µM for the synthetic peptide γ_31-45_*Pv*D_1_^++^ for *S. cerevisiae*. The mechanism of action underlying the death of these yeasts when exposed to these synergistic combinations involves several processes, including the loss of cell wall integrity, impairment of mitochondrial functionality, DNA condensation, and damage to cell morphology and ultrastructure. The data presented in this study will contribute significantly to the development of new therapies involving combinations of antifungal molecules.

**Supplementary Information:**

The online version contains supplementary material available at https://doi.org/10.1007/s00284-026-05197-2.

## Introduction

In recent decades, despite the emergence of new antifungal medications, including broad-spectrum triazoles and echinocandins designed for prophylaxis, empirical therapy, and targeted treatment, the mortality rates associated with candidemia and other invasive infections have remained unchanged [1]. Historically, *Candida albicans* has been the predominant pathogen; however, the incidence of invasive infections caused by *C. tropicalis* has steadily increased.

The vulnerable populations include transplant recipients, individuals with HIV/AIDS or cancer, patients receiving immunosuppressive therapy, those receiving total parenteral nutrition, and premature infants. Non-*albicans Candida* infections now account for 56.5% of candidemia cases, with *C. glabrata* (33.3%), *C. tropicalis* (20.3%), *C. parapsilosis* (1.4%), and *C. kefyr* (1.4%) being the most common *Candida* species [2].

The repeated use of the same medication for different therapies increases the risk of developing resistant microbial strains. Consequently, escalating doses are necessary, which can potentially result in unwanted side effects [3]. The use of combined therapies is considered an excellent strategy to counteract the emergence of resistant strains. Synergistic effects of therapeutic molecules enable dose reduction offering significant advantages [4].

Discovery of new natural antimicrobial peptides (AMPs) is often time-consuming because of their low yield and high production costs. In addition, natural AMPs may present undesirable characteristics for therapeutic applications such as instability due to degradation by host proteases, low selectivity, low water solubility, hemolytic activity, toxicity to the host, and salt sensitivity [5]. Synthetic antimicrobial peptides have emerged as promising alternatives to overcome these limitations. The synthesis of these peptides is based on a rational design that relates structure to the activity of the molecule. Each amino acid was added sequentially, allowing for specific modifications, analysis, and optimization of the molecules [6].

The combination of the antifungal agent amphotericin B (AmB) is endorsed by the Infectious Diseases Society of America for the treatment of *Candida* infections, owing to the increased risk of resistance. Commonly studied combinations involve pairing a commercial antifungal with a specific inhibitor targeting the protein of interest, such as antifungals combined with Hsp90 or protein kinase C inhibitors [7]. In 2011, Sagaram et al. [8] demonstrated that the synthesis of peptides extending beyond the γ-core motif is capable of improving antifungal activity and that the synthetic peptide (GMA-4 C) derived from the γ-core motif that contains the C-terminus of the MtDef4 defensin, which has cationic and hydrophobic amino acids in its structure, was important for its antifungal activity. In 2021, Toledo et al. [9] explored the correlation between the biological activity and structure of plant defensins. In this study, three new peptides with modifications of charge, hydrophobicity (RR and WR), and chirality (D-RR) were designed and tested against pathogenic yeasts.

In 2019, Mello et al. [10] designed four new peptides on the basis of the γ-core motif of *Phaseolus vulgaris* defensin (*Pv*D_1_). On the basis of this γ-core motif sequence together with parts of the β2 and β3 sheets (from Arg31 to Lys45), a 15-residue peptide was designed whose net positive charge increased by replacing the two aspartic acid (Asp) residues at positions 37 and 38 with two arginine (Arg) residues, giving rise to the amino acid sequence RSGRARRRFRAWATK, called γ_31--45_*Pv*D_1_^++^. More recently, our group published a discovery highlighting the synergistic effects of synthetic antimicrobial peptides and amphotericin B against yeast *C. albicans*. This combination was capable of killing this microorganism at doses of amphotericin B 100 times lower than those used commercially [11].

The main objective of this study was to evaluate the synergistic therapeutic effect of the combination of the synthetic peptide γ_31-45_*Pv*D_1_^++^ with AmB *on C. tropicalis* and *Saccharomyces cerevisiae*, as a valuable model for this study, with the goal of identifying a viable synergistic concentration that contributes to the development of new drugs.

## Materials and Methods

### Microorganisms

*Candida tropicalis* CE017, an environmental isolate recovered from wastewater from a petroleum refinery, and *Saccharomyces cerevisiae* 1038, a culture collection strain widely used as a eukaryotic model organism, were obtained from the Department of Biology, Federal University of Ceará (UFC), Fortaleza, Ceará, Brazil. Throughout this study, both yeast strains were maintained on Sabouraud agar supplemented with 2% glucose (Merck) at the Laboratory of Physiology and Biochemistry of Microorganisms, Center for Biosciences and Biotechnology, State University of Northern Rio de Janeiro (UENF), Campos dos Goytacazes, Rio de Janeiro, Brazil. *C. tropicalis* CE017 and *S. cerevisiae* 1038 correspond to the laboratory identification codes assigned to these strains. No public culture collection accession numbers are available.

### Synthetic Peptides

This study used two peptides designed by Mello [10]. γ_31-45_*Pv*D_1_^++^ consists of 15 amino acid residues with a charge of + 7 (RSGRARRRFRAWATK). AminoTech (São Paulo, Brazil) performed peptide synthesis and conducted quality and purity analyses (≥ 95%) via reversed-phase high-performance liquid chromatography (RP-HPLC) and mass spectrometry. The synthetic peptides were solubilized in water and used for all in vitro assays.

### Antifungal Assay with Synthetic Peptides

For yeasts, aliquots were taken from plates containing grown colonies and placed in new Petri dishes containing Sabouraud agar (10 g/L peptone, 2 g/L glucose, and 20 g/L agar) (Merck) with striations in the middle. The new plates were incubated in an oven at 30 °C for 24 h. After growth in a Drigalski loop, the cells were removed and homogenized in 10 mL Sabouraud broth (10 g/L peptone, 2 g/L glucose) (Merck) for quantification in a Neubauer chamber. (Labor Optik), using an optical microscope (Axiovision A2, Zeiss). Yeast cells (1 × 10^4^ cells mL^-1^) were incubated in Sabouraud broth containing pre-established concentrations of synthetic peptides and amphotericin B, which ranged from 1.56 µM to 200 µM. The assay was performed in cell culture microplates (96 wells) at 30 °C for 24 h. Cell growth was determined by optical density, which was monitored at 24 h in a microplate reader at a wavelength of 620 nm. Each test was performed in triplicate. The MIC_100_ was determined as the minimum inhibitory concentration required to completely inhibit fungal growth, and visual interpretation was used for assessment. On the other hand, the MIC_50_ represents the peptide concentration in µM that resulted in 50% inhibition of fungal growth, and it was estimated via nonlinear regression analysis [12].

### Checkerboard Assay

The interaction between Amphotericin B (AmB) and γ_31-45_*Pv*D_1_⁺⁺ was evaluated using the broth microdilution checkerboard method in 96-well microplates, as previously described with minor modifications [31]. Serial two-fold dilutions of AmB (ranging from 0.006 to 1.0 µM) were prepared along the horizontal axis of the microplate, while serial two-fold dilutions of γ_31-45_*Pv*D_1_^⁺⁺^ (ranging from 0.195 to 50 µM) were prepared along the vertical axis. Each well contained a unique combination of both agents in Sabouraud broth.

Yeast suspensions were adjusted to 1 × 10⁴ cells mL⁻¹ and added to each well to a final volume of 100 µL. Control wells containing cells without treatment and wells containing each compound alone were included. Plates were incubated at 30 °C for 24 h under static conditions. Fungal growth was determined by measuring optical density at 620 nm using a microplate reader.

The Fractional Inhibitory Concentration Index (FICI) was calculated according to the following formula: FICI = (MIC of AmB in combination / MIC of AmB alone) + (MIC of peptide in combination / MIC of peptide alone). The interactions were interpreted as follows:

- FICI ≤ 0.5, synergistic;
- 0.5 < FICI ≤ 1.0, additive;
- 1.0 < FICI ≤ 4.0, indifferent;
- FICI > 4.0, antagonistic.

All assays were performed in triplicate in three independent experiments.

### Cell Death Monitoring Assay and Membrane Permeabilization

Cell death was monitored via propidium iodide (PI) staining and plasma membrane permeabilization was assessed via SYTOX Green uptake following previously described methodologies [13]. The yeast cells were incubated with the indicated treatments for 24 h. After incubation, a 100 µL aliquot of each yeast cell suspension was treated with 1 µg/mL PI and 0.2 µM SYTOX Green for 15 min at 30 °C. The cells were then analyzed using a DIC optical microscope (Axiovision 4, Zeiss) equipped with fluorescent filters for PI detection (excitation wavelength: 561 nm; emission wavelength: 630 nm) and fluorescein detection (excitation wavelength: 450–490 nm; emission wavelength: 500 nm).

### Analysis of Vacuolar Membranes

Vacuolar mapping of *C. tropicalis* and *S. cerevisiae* cells was performed using an FM4-64 probe. The yeast cells were subjected to the respective conditions for 24 h. A 100 µL aliquot of each yeast cell suspension was subsequently incubated with 5 µL of FM4-64 for 1 h at 30 °C [14]. The cells were then analyzed using an AxioVision version 4.0, DIC optical microscope (Zeiss) equipped with a 581 nm excitation filter and a 644 nm emission filter.

### Mitochondrial Function Assays

Mitochondrial functionality was evaluated by light fluorescence microscopy with a MitoTracker Red FM fluorescent probe (Thermo Fisher Scientific). After 24 h of incubation, the cells from *C. tropicalis* and *S. cerevisiae* were incubated with 200 nM MitoTracker Red FM for 30 min at 25 °C. The cells were then observed under an optical microscope (Axioplan. A2, Zeiss) attached to an AxioCAM MRc5 camera (Zeiss), and the images were analyzed using AxioVision version 4.0 software (Zeiss) equipped with a 581 nm excitation filter and a 644 nm emission filter. The cells were then heated in the presence of 300 µM acetic acid for 15 min. The experiment was repeated thrice. Triton X-100 was used as a control for loss of cellular membrane integrity.

The activity of mitochondrial dehydrogenase enzymes was also investigated. Yeasts were incubated with 10 µL of 2-(4-iodophenyl)-3-(4-nitrophenyl)-5-(2,4-disulfophenyl)-2 H-tetrazolium, monosodium salt (WST-1) (Sigma‒Aldrich Co.) and 0.24 mM duraquinone (Sigma‒Aldrich Co.) under aseptic conditions. The assays were performed in triplicate. The absorbance of the formazan crystals in the mixture was measured at 450 nm using a microplate reader (EZ Read 400) [15]. The microscopic analyses were performed as previously described for evaluating fungal morphology, membrane integrity, and cellular damage in yeast models [16, 18]. The specificity of mitochondrial impairment was supported by complementary analyses using MitoTracker Red FM staining and transmission electron microscopy.

### Detection of Chromatin Condensation

Nuclear staining with 4’,6-diamidino-2-phenylindole (DAPI; Sigma‒Aldrich) was performed as previously described [16]. The synergistic conditions and controls were transferred to 96-well cell culture plates and incubated for 24 h. A relatively high cell density was used in this assay to allow microscopic visualization of cells. For nuclear staining, after incubation, the cells were washed with PBS, incubated with 4 µg/mL DAPI in PBS for 10 min, rinsed three times with PBS and then visualized under a DIC epifluorescence microscope (Axio Imager. A2; Zeiss) equipped with a fluorescence filter (excitation, 365 nm; emission, 397 nm). The experiment was repeated thrice. The microscopic analyses were performed as previously described for evaluating fungal morphology, membrane integrity, and cellular damage in yeast models [16, 18].

### Detection of Wall Integrity

To assess the integrity of the cell wall, we used the fluorescent probe Calcofluor White (Sigma-Aldrich). After the samples were subjected to synergistic conditions for 24 h, 100 µL portions of the solution were extracted. This portion was subsequently mixed with 10 µL of Calcofluor White and incubated for 5 min at 30 °C in the dark. Following the aforementioned duration, the cells were examined via a light microscope (Axioplan. A2, Zeiss) coupled to an AxioCAM MRc5 camera (Zeiss). The acquired images were subsequently processed via AxioVision version 4.0 software (Zeiss) equipped with a fluorescence filter set (excitation 365 nm; emission 397 nm). To ensure reliable results, the experiment was replicated three times [11].

### Scanning Electron Microscopy Analysis

The cells were washed thrice with PBS and fixed for 48 h in a solution composed of 2.5% glutaraldehyde, 4.0% paraformaldehyde, 0.05 M sodium cacodylate buffer (pH 7.2), and 0.5 mM calcium chloride. Next, three 30 min washes were performed with the same buffer, and the cells were placed under coverslips covered with 0.1% poly-d-lysine, followed by three 15 min washes with the same buffer. The samples were post-fixed in 1% osmium tetroxide for 1 h and washed three times for 15 min with the same buffer. After serial dehydration with increasing ethanol concentration, the samples were subjected to critical point drying using a Bal-Tec Critical Point Dryer CPD 030 apparatus. The dried coverslips were subsequently fixed with carbon adhesive tape on appropriate supports and metallized with a thin layer of 20 nm gold via a Bal-Tec Sputter Coater SCD 050. The samples were observed and documented using a ZEISS DSEM 962 scanning electron microscope at 25 kV. Ultrastructural analysis was conducted following previously established protocols for evaluating fungal cell morphology and structural damage [17, 18].

### Ultrastructure Analysis

For analysis of the ultrastructure, the synergistic conditions were fixed for 1 h in a solution containing 2.5% glutaraldehyde and 4% freshly prepared formaldehyde in 0.1 mol/L sodium cacodylate buffer, pH 7.4. After these procedures, the cells were washed twice with phosphate-buffered saline (PBS) for 10 min and post-fixed for 1 h in the dark with a solution containing 1% osmium tetroxide (OsO4) and 1.6% ferrocyanide in 0.1 M sodium cacodylate buffer. The cells were subsequently washed in the same buffer, dehydrated in acetone (30%, 50%, 70%, 90%, 100%, and 100% superdry), and embedded in Epon. Ultrathin sections were stained with uranyl acetate and lead citrate and observed under a JEOL JEM 1400 Plus transmission electron microscope [17].

### Statistical Analysis

All quantitative experiments were performed using three independent biological replicates, each with three technical replicates. Data are presented as the mean ± standard deviation (SD). Statistical analyses were performed using GraphPad Prism version 8.0.2 for Windows (GraphPad Software, San Diego, CA, USA). Data were analyzed using one-way analysis of variance (ANOVA), followed by Tukey’s multiple comparison test. Different letters indicate statistically significant differences among treatments (*p* < 0.05). Groups sharing the same letter are not significantly different.

## Results

### Effects of Synthetic Peptides and Amphotericin B

Table 1 shows the concentration of AmB required to completely (100%) or partially (50%) inhibit yeast growth. Specifically, for *C. tropicalis* and *S. cerevisiae*, inhibitory concentrations of 0.58 µM and 1.17 µM and 0.195 µM and 0.39 µM, respectively, were observed. Additionally, the synthetic peptide γ_31-45_*Pv*D_1_^++^ impeded 50% of the fungal growth of both *C. tropicalis* and *S. cerevisiae* at concentrations of 3.12 µM and 25 µM, respectively.

**Table 1:** Minimum inhibitory concentration (µM) of synthetic peptide and AmB

	AmBMIC_100_ (µM)	AmBMIC_50_ (µM)	γ_31−45_PvD_1_^++^MIC_50_ (µM)
*Candida tropicalis*	0.58	0.195	3.12
*Saccharomyces cerevisiae*	1.17	0.39	25

### Checkerboard

In our quest for synergistic therapeutic combinations, using a checkerboard assay, we identified synergistic conditions for both *C. tropicalis* and *S. cerevisiae*. Table 2 outlines the concentrations of AmB combined with γ_31-45_*Pv*D_1_^++^ and AmB. Notably, synergistic combinations of 0.048 µM AmB, 0.78 µM γ_31-45_*Pv*D_1_^++^ 0.048 µM AmB and 0.39 µM γ_31-45_*Pv*D_1_^++^ were observed, and synergistic concentrations of 0.048 µM AmB and 0.39 µM γ_31-45_*Pv*D_1_^++^ were used for tests to determine the mechanism of action via electron microscopy.

The lowest concentration was used for the action mechanism and electron microscopy experiments. For the yeast *S. cerevisiae*, the identified combinations with either additive or indifferent effects were as follows: 0.39 µM AmB paired with 6.25 µM γ_31-45_*Pv*D_1_^++^, 0.39 µM AmB paired with 12.5 µM γ_31-45_*Pv*D_1_^++^, and 0.39 µM AmB paired with 3.12 µM γ_31-45_*Pv*D_1_^++^. Notably, a synergistic effect of 0.097 µM AmB and 0.78 µM γ_31-45_*Pv*D_1_^++^ was detected, as shown in Table 3. The combination that showed synergism was used in the mechanism of action and electron microscopy experiments.

**Table 2:** Combined activity of the bioinspired peptide and AmB against *C. tropicalis*

DRUG A	DRUG B	FIC A (µM)	FIC B (µM)	∑ FIC	ACTION
AmB	γ_31−45_PvD_1_^++^	0.048/0.195	3.12/3.12	1.24	Additive or Indifferent
	γ_31−45_PvD_1_^++^	0.048/0.195	1.56/3.12	0.74	Additive or Indifferent
	γ_31−45_*Pv*D_1_^++^	0.048/0.195	0.78/3.12	0.49	Synergism
	γ_31−45_*Pv*D_1_^++^	0.048/0.195	0.39/3.12	0.36	Synergism

**Table 3:** Combined activity of the bioinspired peptide and AmB against *S. cerevisiae*

DRUG A	DRUG B	FIC A (µM)	FIC B (µM)	∑ FIC	ACTION
AmB	γ_31−45_PvD_1_^++^	0.39/0.39	12.5/25	1.5	Additive or Indifferent
	γ_31−45_PvD_1_^++^	0.39/0.39	6.25/25	1.25	Additive or Indifferent
	γ_31−45_*Pv*D_1_^++^	0.39/0.39	3.12/25	1.12	Additive or Indifferent
	γ_31−45_*Pv*D_1_^++^	0.097/0.39	0.78/25	0.27	Synergism

### Cell Death Monitoring Assay and Membrane Permeabilization

**Fig. 1:**
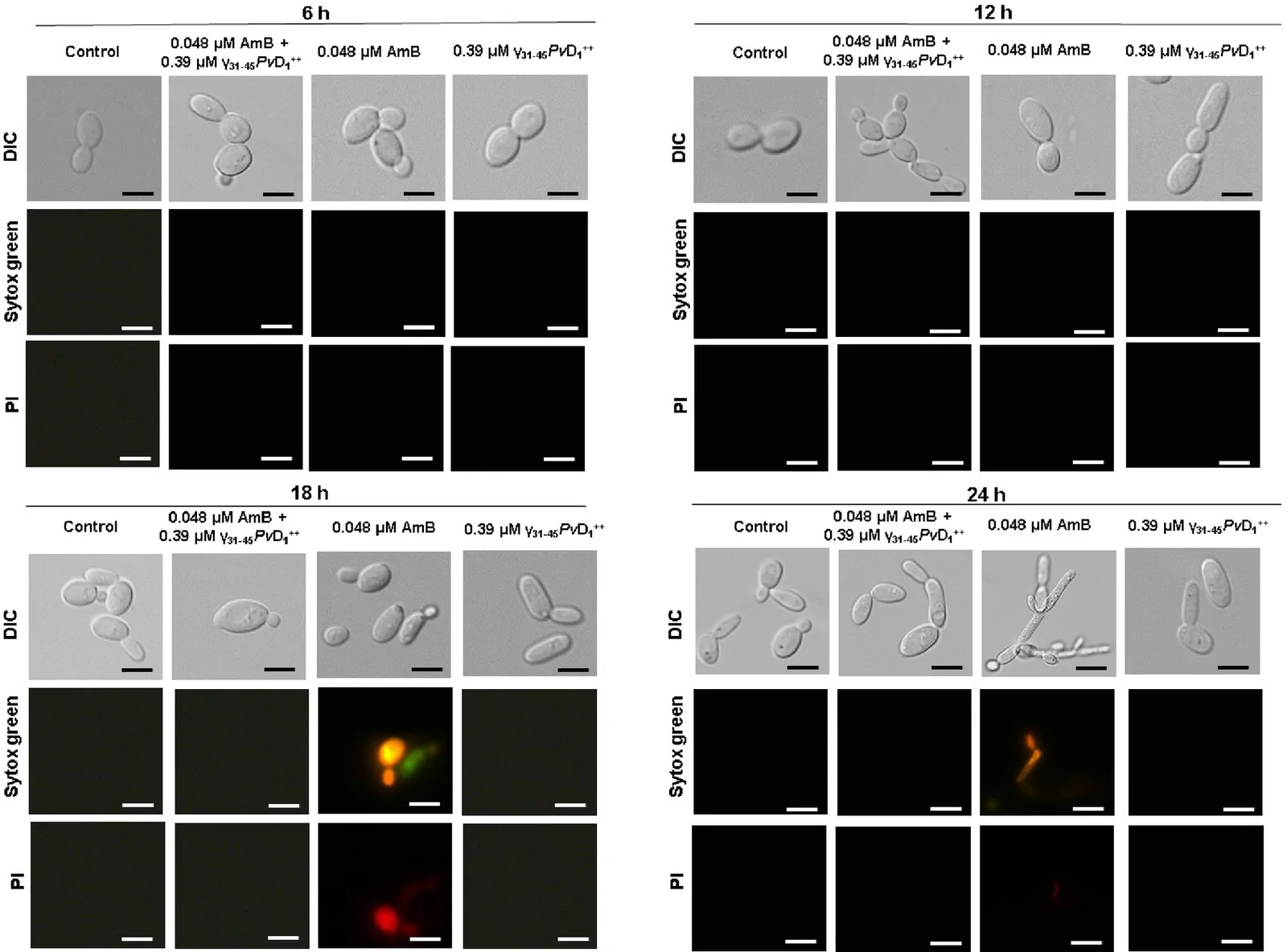
Cell death monitoring assay and membrane permeabilization: Fluorescence microscopy images of *C. tropicalis* cells following a membrane permeabilization assay using the fluorescent probe SYTOX Green. The cells were exposed to the synthetic peptide and AmB alone or in combination for 6, 12, 18, or 24 h and then analyzed for membrane permeabilization. All the samples were also treated with propidium iodide. Bars = 20 µm

We analyzed the mechanism of action of the synergistic combinations identified in the yeasts used in this study. Using the SYTOX Green probe, we evaluated whether the tested concentrations were capable of causing permeabilized the cell membrane. In addition, we employed a propidium iodide (PI) assay to verify whether the necrotic pathway was activated. Figure 1 presents the results of these analyses on *Candida tropicalis* cells. During follow-up periods of 6, 12, 18, and 24 h of incubation, at the AmB concentration used and only after 18 h of incubation, it was possible to observe the labeling of both types of fluorescence. No labeling was detected under other conditions, including the synergistic combination. These results suggest that the mechanism responsible for the death of this microorganism does not involve necrosis or permeabilization of the plasma membrane.

**Fig. 2:**
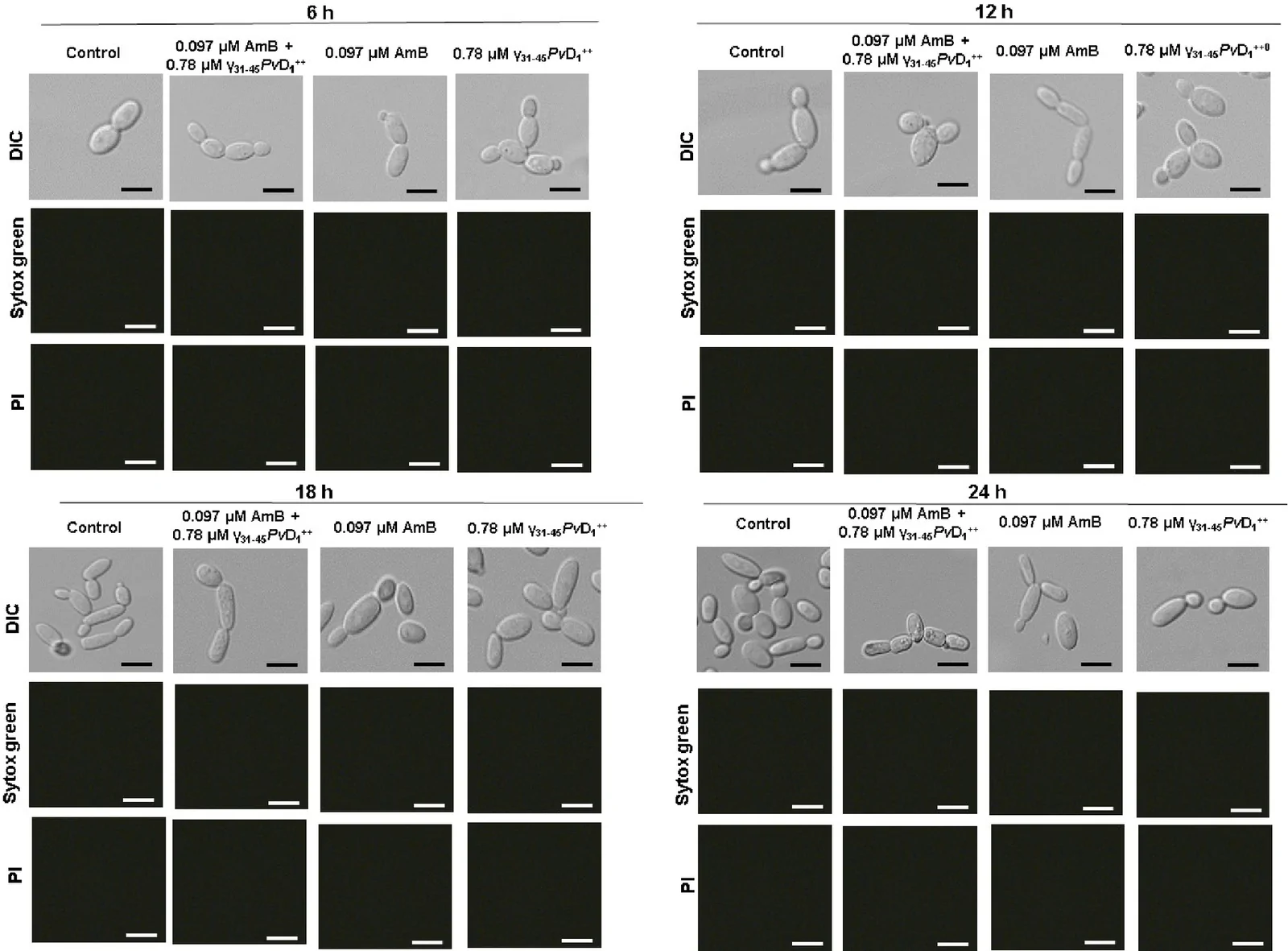
Cell death monitoring assay and membrane permeabilization: Fluorescence microscopy images of *S. cerevisiae* cells following a membrane permeabilization assay using the fluorescent probe SYTOX Green. The cells were exposed to the synthetic peptide and AmB alone or in combination for 6, 12, 18, or 24 h and then analyzed for membrane permeabilization. All the samples were also treated with propidium iodide (PI). Bars = 20 µm

The results of these analyses for the yeast *Saccharomyces cerevisiae* are shown in Fig. 2. Probe labeling was not observed at any of the analyzed time points (6, 12, 18, and 24 h). These findings suggest that none of the treatments, including the synergistic combination, affected membrane permeabilization or activation of the necrosis pathway in this yeast.

### Analysis of Vacuolar Membranes and Mitochondrial Functionality in *C. tropicalis* and *S. cerevisiae*

**Fig. 3:**
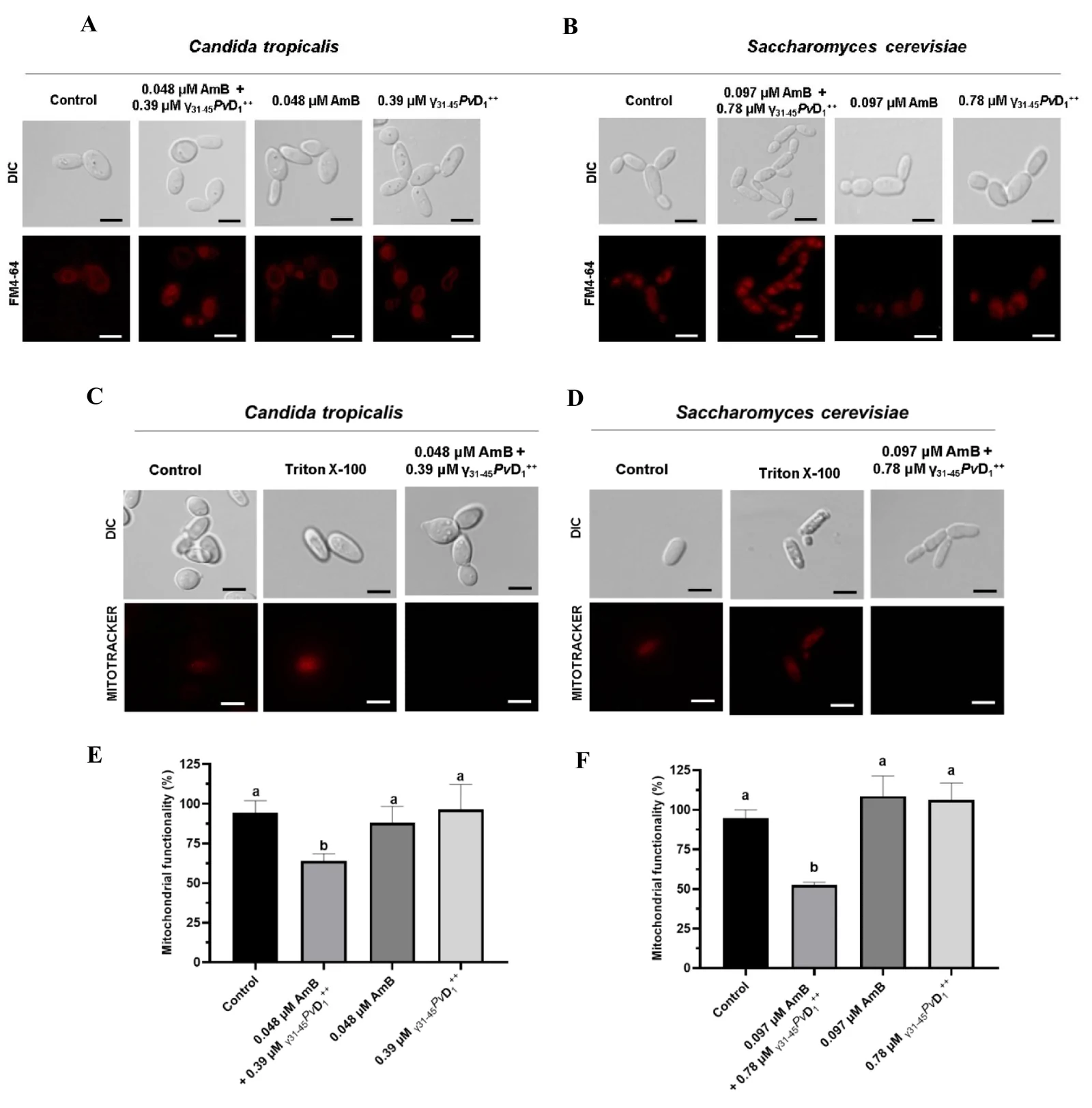
Vacuolar mapping via the FM4-64 probe. *C. tropicalis* (**A**) and *S. cerevisiae* (**B**) control cells, AmB cells and synthetic peptides alone or in combination for 24 h and then treated with 5 µM FM4-64 for 60 min at 30 °C. The control was treated with FM4-64 alone. Bars = 20 μm. The Mitochondrial functionality, fluorescence microscopy images of *C. tropicalis* (**C**) and *S. cerevisiae* (**D**) cells treated with the synergistic combination of _γ31–45_*Pv*D_1_^++^ and amphotericin B (AmB), the corresponding individual treatments, untreated control, and 1% Triton X-100 (control cellular membrane integrity), incubated for 24 h with 200 nM MitoTracker. MitoTracker is a live-cell probe, and cells were imaged in suspension without fixation or adherence-promoting treatments. Minor repositioning of cells during image acquisition may result in slight spatial differences between DIC and fluorescence images. Panels (**E**) and (**F**) show mitochondrial dehydrogenase activity measured using the WST-1 assay. Data are presented as the mean ± SD of three independent biological experiments, each performed in triplicate. Statistical analysis was performed using one-way ANOVA, followed by Tukey’s multiple comparison test. Different letters indicate statistically significant differences among treatments (*p* < 0.05). Groups sharing the same letter are not significantly different

The effects of synergistic combinations of *Candida tropicalis* and *Saccharomyces cerevisiae* on vacuolar membranes were evaluated using an FM4-64 probe. This probe is lipophilic and cannot cross cell membranes, but intercalates in the plasma membrane and is internalized by cells by endocytosis, allowing the labeling of vacuolar membranes. After incubating the *C. tropicalis* and *S. cerevisiae* cells for 24 h in the presence or absence of the synergistic combinations, the cells were reincubated with 5 µM FM4-64 probe for 60 min at 30 °C. Figure 3A and B shows the results of this analysis, which revealed that the FM4-64 probe labeled the vacuole membrane, resulting in a ring staining pattern in all cells of the control and treatment groups, and a similar pattern in *C. tropicalis* cells. However, in *S. cerevisiae*, more vacuoles were observed in the samples treated with the synergistic combination than in the control samples and samples treated with both the synthetic peptide and AmB.

The results indicated that the combination of the synthetic peptide γ_31-45_*Pv*D_1_^⁺⁺^ and AmB at synergistic concentrations affected mitochondrial functionality in *C. tropicalis* (Fig. 3C) and *S. cerevisiae* cells (Fig. 3D). Synergistic concentrations of 0.39 µM **+** γ_31-45_*Pv*D_1_^⁺⁺^ and 0.048 µM AmB for *C. tropicalis*, as well as 0.78 µM γ_31-45_*Pv*D_1_^⁺⁺^ and 0.097 µM AmB for *S. cerevisiae*, resulted in a notable alteration in mitochondrial functionality. In contrast, when the peptide or AmB was used individually at the same concentrations as those used in the synergistic combinations, mitochondrial functionality remained comparable to that observed in the untreated controls. Triton X-100 was used as a control for loss of cellular membrane integrity. Therefore, the MitoTracker results were interpreted as evidence of alterations in mitochondrial functionality associated with the synergistic treatments, rather than as definitive evidence of mitochondrial depolarization. Minor differences in cell positioning between the DIC and fluorescence images may have occurred due to cell movement during live-cell imaging and should therefore be considered when comparing the corresponding panels.

Cellular metabolic/dehydrogenase activity was evaluated using the WST-1 assay, which measures the conversion of WST-1 to formazan by cellular dehydrogenases. In *C. tropicalis* cells, the synergistic combination decreased dehydrogenase activity by 36% (Fig. 3E), whereas in *S. cerevisiae* cells, a 48% decrease in dehydrogenase activity was observed (Fig. 3F). These results indicate that the synergistic combinations significantly affected cellular metabolic activity in both *C. tropicalis* and *S. cerevisiae*. Given that mitochondrial dehydrogenases contribute to WST-1 reduction, these findings are consistent with impaired mitochondrial activity. However, the WST-1 assay does not provide a specific measure of mitochondrial dysfunction. The reduction in WST-1 activity, together with the alterations in mitochondrial membrane potential observed by MitoTracker staining and the ultrastructural changes observed by transmission electron microscopy, supports an association between the synergistic treatments and altered mitochondrial activity.

### Chromatin Condensation and Detection of Wall Integrity in *C. tropicalis* and *S. cerevisiae*

**Fig. 4:**
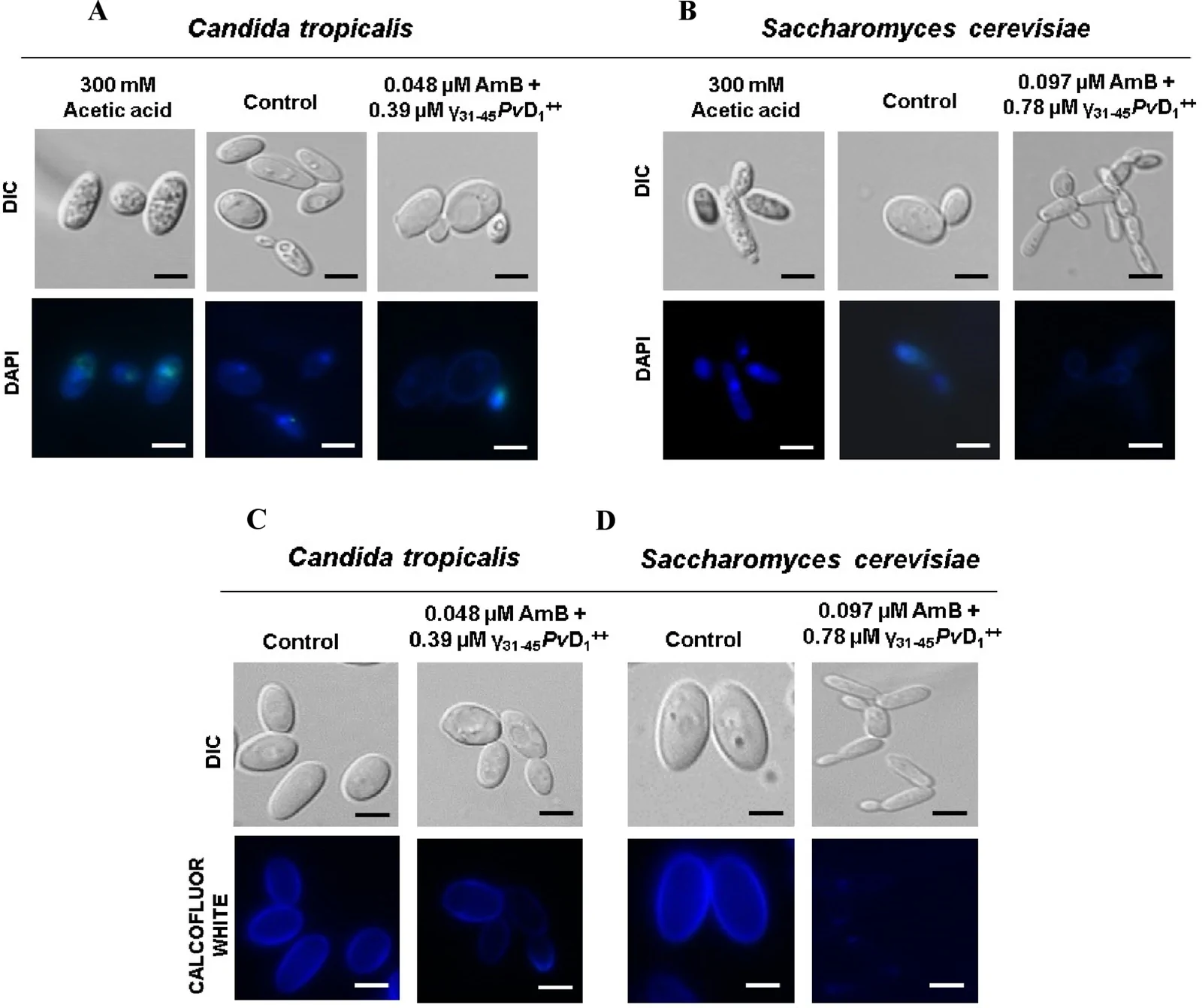
Chromatin condensation assay images of *C. tropicalis* (**A**) and *S. cerevisiae* (**B**)cells after treatment with γ_31-45_*Pv*D_1_^++^ in combination with AmB for 24 h; control (untreated) and 300 mM acetic acid (positive control) were used. To visualize the nuclei, DAPI was used. The wall integrity of *C. tropicalis* (**C**) and *S. cerevisiae* (**D**) cells after treatment with γ_31-45_*Pv*D_1_^++^ in combination with AmB for 24 h; control (untreated). The wall integrity of the cells was assessed via a Calcofluor white probe. Bars = 20 μm

The synergistic combinations of 0.39 µM γ_31-45_*Pv*D_1_^⁺⁺^ and 0.048 µM AmB for *C. tropicalis*, as well as 0.78 µM γ_31-45_*Pv*D_1_^⁺⁺^ and 0.097 µM AmB for *S. cerevisiae*, induced alterations in the nuclear staining pattern. In control cells, labeling with 4’,6-diamidino-2-phenylindole (DAPI) resulted in intense and punctate fluorescence, consistent with normal nuclear staining. In contrast, cells treated with the synergistic combinations of both yeasts exhibited an altered DAPI staining pattern, characterized by a reduction or absence of the punctate fluorescence observed in control cells (Fig. 4A and B). These findings are consistent with nuclear alterations induced by the synergistic treatments.

Analysis of the integrity of the cell walls of *C. tropicalis* (Fig. 4C) and *S. cerevisiae* (Fig. 4D) was carried out after 24 h in the presence of synergistic combinations of the synthetic peptides γ_31-45_*Pv*D_1_^++^ and AmB. At control concentrations, we observed cells that were strongly stained with calcofluor white, indicating cell wall integrity. The resulting blue fluorescence indicates the presence of an intact cell wall, as clearly represented in Fig. 4C and D. However, after treatment with the synergistic combinations, intense labeling was not observed, suggesting a loss of cell wall integrity and possibly a decrease in cell viability, especially in *S. cerevisiae*.

### Scanning Electron Microscopy

**Fig. 5:**
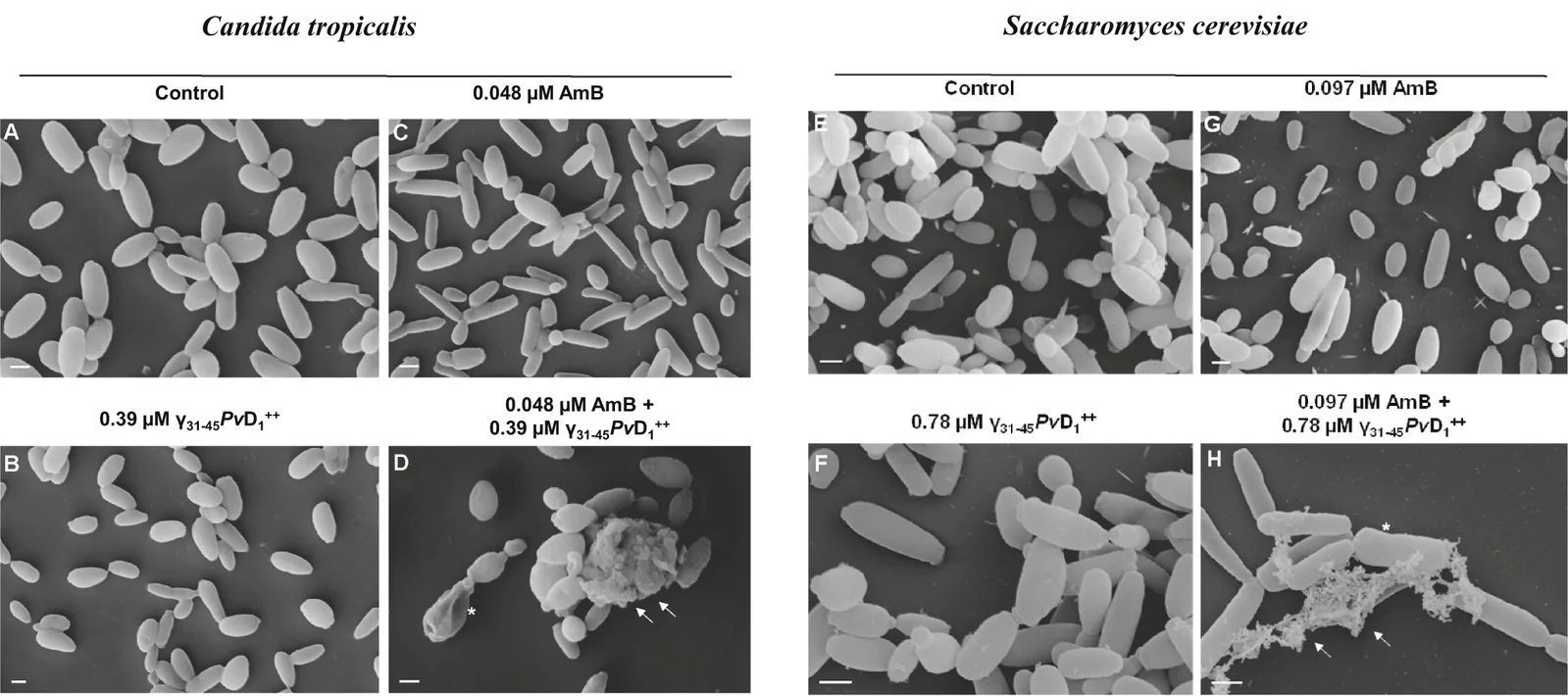
Scanning electron microscopy (SEM) images showing changes in the cell surfaces of *C. tropicalis* (**A**-**D**) and *S. cerevisiae* (**E**-**H**). Arrows indicate loss of cytoplasmic material and asterisks denote changes in the membrane surface. Scale bars = 1 μm

Scanning electron microscopy (SEM) was employed to evaluate the surface damage to *C. tropicalis* (Fig. 5A, B, C, and D) and *S. cerevisiae* (Fig. 5E, F, G, and H) following all treatments. For *C. tropicalis*, the control cells (Fig. 5A) did not show any changes in surface area. Treatment with AmB alone (Fig. 5C) resulted in cell elongation, but no major changes in the cell surface; after treatment with the individual synthetic peptides (Fig. 5B), the cells were similar to those of the control. However, the combination of both molecules (Fig. 5D) caused some damage to cells, including pronounced roughness and evident cell lysis. For *S. cerevisiae*, the control cells (Fig. 5E) showed no surface damage or alterations; they appeared as oval-shaped cells without cracks or scars. Treatment with the synthetic peptide (Fig. 5F) or AmB (Fig. 5G) alone resulted in only minor damage, such as wrinkles and slight changes in cell morphology, which closely resembled those of the control. In contrast, the combination of both molecules (Fig. 5H) caused severe damage to the cells, including squamous and rod-shaped cells with pronounced roughness, significant morphological changes, and evident signs of cell lysis, leading to cytoplasmic loss. The ultrastructural characteristics of *C. tropicalis* (Figure Supplementary 1 A-D) and* S. cerevisiae* (Fig. S1E-H) cells under all treatment and control conditions are shown in detail. In control *C. tropicalis* cells (Fig. S1A), the cell wall and plasma membrane remained intact, as did organelles such as the nucleus, mitochondria, and vacuoles. After treatment with the synthetic peptide (Fig. S1C) or AmB (Fig. S1B) alone, the cells and organelles remained similar to those of the control. However, in the cells treated with the synergistic combination (Fig. S1D), the genetic material and mitochondria were significantly damaged.

Figure S1 shows striking differences in cellular components, such as the cell wall, plasma membrane, and some organelles. The control cells (Figure S1E) presented a uniform surface with no visible rupture. When treated with the synthetic peptide (Figure S1G) or AmB (Figure S1F) alone, the cells closely resembled those of the control. In particular, treatment with the synthetic peptide increased the number of vacuoles, indicating a possible defense response against the bioinspired peptide in *S. cerevisiae*. In contrast, under synergistic conditions (Figure S1H), *S. cerevisiae* cells presented severe impairments in plasma membrane integrity, cell wall structure, and cytoplasmic organization, indicating cellular extravasation.

## Discussion

Natural plant antimicrobial peptides (AMPs) are promising therapeutic agents for the treatment of various infections. However, these methods have drawbacks, such as susceptibility to proteolysis and high costs associated with isolation and purification. The development of synthetic antimicrobial peptides based on natural molecules offers a viable solution to overcome these limitations. Synthetic peptides can retain the antimicrobial efficacy of their natural counterparts, while potentially mitigating issues such as toxicity to mammalian cells and reducing the likelihood of antimicrobial resistance owing to their distinct mechanisms of action [18, 19]. The present study demonstrates that the bioinspired peptide γ_31-45_*Pv*D_1_^⁺⁺^ enhances the antifungal activity of Amphotericin B (AmB) against *Candida tropicalis* and *Saccharomyces cerevisiae*, resulting in synergistic interactions that affect membrane integrity, mitochondrial function, and nuclear organization. *S. cerevisiae* was included in this study as a well-established model yeast to investigate cellular and mitochondrial responses to the synergistic treatment, rather than as a model of human cytotoxicity or a clinically relevant pathogenic yeast. These findings provide mechanistic insight into peptide–polyene combinatorial strategies and reinforce the potential of antimicrobial peptides as adjuvants in antifungal therapy.

Although the present work extends our previous findings in *Candida albicans*, the mechanistic outcomes observed in *C. tropicalis* and *S. cerevisiae* reveal important species-dependent differences. Notably, unlike C. albicans, neither *C. tropicalis* nor *S. cerevisiae* exhibited plasma membrane permeabilization (SYTOX Green uptake) or propidium iodide staining under synergistic conditions, suggesting that the fungicidal synergy between amphotericin B and γ_31-45_*Pv*D_1_^++^ is not strictly dependent on membrane pore formation. These differences may reflect variations in membrane lipid composition, ergosterol content and distribution, and/or cell wall architecture across yeast species [20]. Nevertheless, the synergistic treatment consistently triggered mitochondrial dysfunction, chromatin damage, cell wall integrity loss, and severe morphological and ultrastructural alterations, supporting mitochondrial impairment and cell wall destabilization as conserved downstream events of this combination. Differences in membrane sterol composition and regulation between pathogenic *Candida* species and *S. cerevisiae* have been documented. For example, variations in plasma membrane lipid composition, including ergosterol levels, influence antifungal susceptibility across yeast species [21]. Additionally, ergosterol biosynthesis and regulation differ among Candida spp., potentially affecting membrane fluidity, permeability and drug responses. Alterations in key ergosterol-related genes also lead to significant changes in sterol composition and membrane properties in *Candida* [22].

Rational design of synthetic peptides based on sequences from natural proteins has gained traction owing to several advantages. These synthetic peptides can be meticulously designed to increase their efficacy while minimizing their potential side effects. Moreover, multiple functionalities could be incorporated into the same peptide sequence. Several online servers facilitate peptide design [23], enabling the creation of peptides with characteristics that may not exist in proteins that serve as models [24].

In this study, we investigated the synergistic effects of a synthetic peptide based on the γ-core motif of *P. vulgaris* seed defensin, known as γ_31-45_*Pv*D_1_^++^, in combination with the widely used drug amphotericin B for combating *C. tropicalis* and using *S. cerevisiae* as a model. *S. cerevisiae* is widely used as a model for the cells of more complex organisms, such as humans. This yeast is the first eukaryote whose complete genome has been sequenced [25]. The main characteristics that make it an excellent model include its short life cycle, ease of manipulation, well-annotated genome, wide range of molecular tools, and conservation of basic eukaryotic biological and biochemical pathways [26].

Recently, our research group discovered synergistic interactions between the synthetic peptides γ_31-45_*Pv*D_1_^++^ and AmB against the yeast *C. albicans*. This combination exhibited a potent fungicidal effect characterized by increased membrane permeability, impaired mitochondrial function, and DNA degradation [11].

Although synergy was clearly detected by checkerboard assays and confirmed by multiple downstream phenotypes, the present study does not directly determine whether amphotericin B or γ_31-45_*Pv*D_1_^++^ triggers the first molecular event in the target cells. Time-course microscopy using SYTOX Green and propidium iodide (6–24 h) did not indicate plasma membrane permeabilization under synergistic conditions in either *C. tropicalis* or *S. cerevisiae*, suggesting that membrane pore formation is not an early hallmark of the synergistic interaction in these species. A plausible model is that amphotericin B initially binds ergosterol and perturbs membrane organization, which may facilitate peptide interaction and promote intracellular stress responses leading to mitochondrial dysfunction and cell wall destabilization. Alternatively, γ_31-45_*Pv*D_1_^++^ may first interact with cell wall components and indirectly increase amphotericin B access to ergosterol-rich membrane domains. AmB activity is closely associated with its interaction with ergosterol in fungal membranes, and this interaction is considered an important determinant of its antifungal activity. In the present study, the synergistic interaction between γ_31-45_*Pv*D_1_^++^ and AmB may suggest that the peptide facilitates or enhances the action of AmB at the fungal membrane. However, the present study did not directly assess whether γ_31-45_*Pv*D_1_^++^ enhances AmB-mediated ergosterol extraction or alters sterol distribution or accessibility. Therefore, enhanced sterol extraction should be considered a possible mechanism underlying the observed synergistic interaction rather than a demonstrated mechanism. Further experiments, such as filipin staining, quantitative ergosterol analysis, or sterol competition assays, would be necessary to determine whether γ_31-45_*Pv*D_1_^++^ modulates ergosterol accessibility or enhances the interaction of AmB with membrane sterols. Further studies employing early time points and biophysical assays will be required to clarify the temporal sequence of events. We initiated our quest for a new combined therapeutic option against clinically significant yeasts by determining the MIC_50_ values of a synthetic peptide and AmB. We subsequently conducted a checkerboard test to identify the combinations that exhibited synergistic effects. Synergistic combinations for the development of combination therapies In this study, the synergisti were identified for both the yeasts using the checkerboard method. Consequently, synergistic concentrations of 0.048 µM AmB + 0.39 µM γ_31-45_*Pv*D_1_^++^ for *C. tropicalis* (Table 2) and 0.097 µM AmB + 0.78 µM γ_31-45_*Pv*D_1_^++^ for *S. cerevisiae* (Table 3) were used to determine the mechanism of action involved in the death of each yeast studied.

An additional aspect that reinforces the translational relevance of the present findings is the marked reduction in amphotericin B concentrations achieved through synergistic combinations with γ_31-45_*Pv*D_1_^++^. Amphotericin B remains one of the most effective antifungal agents available; however, its clinical use is limited by dose-dependent nephrotoxicity and infusion-related adverse effects. Therefore, strategies capable of maintaining antifungal efficacy while reducing the required amphotericin B concentration may contribute to improved therapeutic safety and tolerability. Although the synergistic concentrations identified in this study were determined exclusively under in vitro conditions, they provide a rationale for further pharmacokinetic/pharmacodynamic (PK/PD) investigations in animal models and clinically relevant fungal isolates. The selectivity profile γ_31-45_*Pv*D_1_^⁺⁺^ is also an important consideration for the development of peptide-based combination therapies. Previous studies from our research group [10] demonstrated that this bioinspired peptide exhibits low hemolytic activity and limited toxicity toward mammalian cells at antifungal concentrations, suggesting a favorable selectivity profile for further investigation. However, an important limitation of the present study is that cytotoxicity was not evaluated under combined amphotericin B and γ_31-45_*Pv*D_1_^⁺⁺^ treatment conditions. Because the proposed therapeutic advantage of this strategy relies on the synergistic use of both molecules, additional studies should include hemolysis assays and mammalian cell viability analyses under co-treatment conditions to determine the selectivity and safety of the combination before any therapeutic conclusions can be established.

A limitation of the present study is that sterol extraction and quantification assays were not performed. Consequently, although our results are consistent with a model in which amphotericin B-mediated membrane perturbation facilitates downstream intracellular damage, including mitochondrial dysfunction and cell wall destabilization, the direct involvement of ergosterol-related membrane alterations remains a mechanistic hypothesis. Future studies employing sterol profiling, membrane biophysical analyses, and early time-point experiments will be important to validate this proposed mechanism and define the temporal sequence of events underlying the synergistic interaction. An important aspect of the present findings is the approximately four-fold reduction in the effective concentration of amphotericin B achieved through synergistic combinations with γ_31-45_*Pv*D_1_^++^ .Amphotericin B remains one of the most effective antifungal agents available for the treatment of invasive fungal infections; however, its clinical use is frequently limited by dose-dependent nephrotoxicity and infusion-related adverse effects. Therefore, strategies capable of enhancing antifungal activity while reducing the required concentration of amphotericin B are of considerable interest.

Nevertheless, the reduction observed in the present study was determined exclusively under in vitro conditions and should not be interpreted as evidence that an equivalent reduction in therapeutic dosage would occur in vivo. The relationship between in vitro synergistic concentrations and clinically effective drug exposure depends on complex pharmacokinetic and pharmacodynamic (PK/PD) factors, tissue distribution, host immune responses, and fungal burden. Consequently, the observed synergistic interaction should be considered as a promising rationale for future translational studies rather than as direct evidence of an immediately applicable therapeutic strategy. Further PK/PD-guided dose-finding studies, toxicity assessments, and in vivo efficacy evaluations will be necessary to determine whether this combination can provide clinically meaningful reductions in amphotericin B exposure while maintaining antifungal efficacy.

The MIC_50_ of γ_31-45_*Pv*D_1_^++^ differed markedly between the two yeasts tested, being 3.12 µM for *C. tropicalis* and 25 µM for *S. cerevisiae* (~ 8-fold difference). This variability is consistent with the fact that susceptibility to cationic antimicrobial peptides is highly species-dependent. A plausible explanation involves differences in cell wall architecture, including the relative abundance and organization of β-glucans, mannoproteins, and chitin, which may affect peptide binding and diffusion through the wall. In addition, variations in plasma membrane lipid composition (e.g., ergosterol and sphingolipids) may influence peptide–membrane interactions and downstream intracellular effects. Differences in peptide uptake, intracellular trafficking, and stress-response pathways may also contribute. Notably, *S. cerevisiae* displayed increased vacuolar structures under synergistic treatment, suggesting activation of adaptive responses and/or sequestration mechanisms that could reduce peptide effectiveness. These intrinsic biological differences likely account for the higher MIC_50_ observed in *S. cerevisiae* compared with the pathogenic yeast *C. tropicalis* [13].

The study of combination therapies involving conventional antifungals and antifungal molecules is a good path for the development of new drugs. Costa et al. 2020 [27], in the search for synergistic concentrations, used etoconazole as a standard for dermatophytes and amphotericin B for yeasts. The essential oil used in this study showed intrinsic activity against dermatophytes, a synergistic effect against all strains of *Tricophyton rubrum* when combined with ketoconazole, and a synergistic effect with amphotericin B against two strains of *C. tropicalis* and one strain of *C. parapsilosis.*

We investigated whether the identified synergistic concentrations could induce membrane permeabilization leading to the death of the tested microorganisms. To assess this, we used SYTOX Green and propidium iodide probes and conducted analyses after 6, 12, 18, and 24 h of incubation. For *C. tropicalis* (Fig. 1), we observed positive staining for both probes at a concentration of 0.048 µM AmB after 18 h of incubation; however, no positive staining was detected under the other conditions. These results indicated that the synergistic combination identified for this yeast strain did not promote membrane permeabilization. For *S. cerevisiae* (Fig. 2), we did not observe positive labeling for either probe at any of the tested time points. This finding indicated that the synergistic combination identified for this yeast strain was also unable to promote membrane permeabilization. *S. cerevisiae* has a complex response mechanism, including the production of compounds for self-protection and activation of heat shock proteins (HSPs) to maintain cellular integrity [28]. Silva et al. analyzed some of these proteins, addressing their structural and molecular characteristics, as well as the protein profiles of industrial strains of *S. cerevisiae* under heat stress. The results showed that temperature variations influenced the protein profiles of these strains, altering their ability to protect the plasma membrane. In the present study, *S. cerevisiae* cells were not subjected to any type of physical stress; therefore, their self-protective mechanisms remained unchanged, explaining the absence of membrane permeabilization in these cells.

To investigate the mechanisms of action involved in the death of yeast treated with the identified synergistic concentrations, we analyzed the vacuoles of both species. Cytoplasmic vacuolization is a well-documented morphological phenomenon that often occurs in mammalian cells after exposure to bacterial or viral pathogens, and is similar to various natural and artificial low-molecular-weight compounds. However, the mechanisms underlying vacuolization in yeast are still being investigated for a more comprehensive understanding. Although vacuolization is typically accompanied by cell death, its specific role in these processes remains unclear [29]. In the present study, we observed significant differences in the number of vacuoles in *S. cerevisiae* between the control and synergistic *treatments*, which were not evident in *C. tropicalis*.

The data for *S. cerevisiae* are shown in Fig. 3B, in which the FM4-64 probe was used to visualize the vacuolar membranes. The number of vacuoles in the cells was greater in the synergistic treatment group than in the control group, suggesting the onset of cell death. Yoshioka (2016) [30] demonstrated that the fungicidal activity of amphotericin B relies on autophagy-dependent targeting of the vacuole during nutrient starvation in *Saccharomyces cerevisiae*. These findings support the idea that amphotericin B undergoes autophagy-mediated trafficking to the intravacuolar lumen, where it interacts with the luminal membrane leaflet, causing severe structural damage. These results may explain the absence of vacuolar damage when cells were treated solely with synthetic peptides.

Mitochondrial function is crucial for cellular processes and development. We investigated whether the synergistic combinations identified in each yeast strain could impair mitochondrial function. Both combinations were capable of damaging mitochondria (Fig. 3C-D).

In 2022, Bezerra et al. [31] studied the effects of combining the synthetic peptides Mo-CBP3-PepI and Mo-CBP3-PepIII (50 µg mL) with the drugs nystatin and itraconazole on the development of *C. albicans* and *C. parapsilosis*, and their results demonstrated that the combination of these molecules significantly increased the endogenous production of reactive oxygen species (ROS), which is directly related to the loss of mitochondrial functionality.

Aguiar et al., 2023 [32] demonstrated that combinations of the drug itraconazole and the synthetic peptides Mo-CBP3-PepII, RcAlb-PepII, RcAlb-PepIII, PepGAT and PepKAA (25 µM) induced the formation of membrane pores and the overaccumulation of ROS in *Cryptococcus neoformans* cells, which is a characteristic of ROS accumulation related to the loss of mitochondrial functionality.

Lucas, et al., 2023 [33] investigated the mechanism of action of three bioinspired peptides, RR (A36,42,44R37,38γ32-46VuDef), D-RR (D-A36,42,44R37,38γ32-46VuDef) and WR (A42,44R37,38W36,39γ32-46VuDef), against the yeasts *C. tropicalis* and *C. albicans*. Among the studied mechanisms, the focus was on mitochondrial functionality, with all peptides demonstrating the ability to cause mitochondrial damage. The results showed that for *C. tropicalis*, the RR and D-RR peptides increased mitochondrial activity by 305.2% and 565.1% after 1 h and 30 min of incubation, respectively. In *C. albicans*, the WR peptide resulted in an increase of 835.9% in mitochondrial activity after 20 min of incubation and 376.4% after 1 h. One of the authors concluded that mitochondrial functionality is one of the main targets of bioinspired peptides, contributing to the death of the yeasts *C. tropicalis* and *C. albicans.* [33]

Another important aspect of antifungal mechanisms involves indirect DNA damage mediated by oxidative stress pathways. Several antifungal agents promote the accumulation of reactive oxygen species (ROS), leading to mitochondrial dysfunction, activation of metacaspase-dependent apoptosis-like pathways, chromatin condensation, and DNA fragmentation in fungal cells [34]. Therefore, this study evaluated the ability of the synergistic combinations identified for each yeast strain to cause DNA degradation. Intense and concentrated nuclear fluorescence was observed in both the control group and groups treated with individual concentrations of the synergistic agents.

This finding strongly suggests preservation of nuclear integrity, as depicted in Fig. 4A-B. However, in synergistic combinations, the absence of nuclear staining suggested significant chromatin condensation or potential leakage of DNA along with intracellular materials. Under synergistic conditions, the altered or absent nuclear staining pattern suggests changes in chromatin organization or nuclear integrity. However, this observation should not be interpreted as indicative of a stronger degree of chromatin condensation compared to acetic acid treatment, as different stressors may activate distinct regulated cell death pathways in yeasts.

The 4’,6’-Diamidino-2-phenylindole (DAPI) was used to evaluate DNA integrity in our study. This compound binds to DNA, is not cell-permeable, and selectively interacts with the minor groove of double-stranded DNA, emitting fluorescence upon binding. By binding to DNA, DAPI facilitates the visualization and assessment of cellular processes involving DNA, such as chromatin condensation and potential leakage of genetic material due to the rupture of the cell wall and plasma membrane. The use of DAPI as a tool in our study proved to be invaluable for understanding the cellular changes induced by synergistic combinations [34].

In 2017, Soares et al. [16] demonstrated that defensin ApDef1 induces cell death in *S. cerevisiae* cells with an MIC of 7.8 µM. Significant cell death was observed after 18 h of exposure to ApDef1, with 98.76% of the cell population dying within 3 h. Incubation with ApDef1 resulted in membrane permeabilization, increased production of endogenous reactive oxygen species (ROS), chromatin condensation, and caspase activation. Similar to our study, the authors used a DAPI probe for chromatin condensation analysis, highlighting its efficacy as a tool for such investigations.

The pursuit of combination therapies involving antifungals that target microbial cell walls has garnered significant interest among scientists seeking innovative therapeutic alternatives. In 2019, the World Health Organization (WHO) established a list aimed at combating resistant pathogens and categorizing them according to the level of threat they pose to public health. Pathogens classified as “urgent threats” include *Candida auris* and certain antibiotic-resistant bacteria. The “serious threats” category encompasses pathogens whose impact on public health necessitates prompt and sustained action. Notably, these included serious infections caused by drug-resistant species within the *Candida* genus, primarily *C. albicans*, followed by *C. tropicalis*, *C. parapsilosis*, and *C. krusei*.

In this study, the effects of synergistic combinations of *C. tropicalis* and *S. cerevisiae* on the integrity of fungal cell walls were assessed using the calcofluor dye. This dye has an affinity for components present in the fungal cell wall, aiding in the observation of structural changes and damage caused by treatments. Figure 4C-D presents the results of these analyses. Blue fluorescence indicates the presence of an intact cell wall, as observed in the control and individual synergistic combinations. Following treatment with synergistic combinations of *C. tropicalis* and *S. cerevisiae*, the intense cell wall markings observed in the control and individual treatments were notably absent. Instead, fragmented markings were observed, indicating loss of cell wall integrity and potentially compromised cell viability. Similar effects were observed by Souza et al. [11] using a synergistic combination of the same synthetic peptide and AmB against *C. albicans*. The synergistic concentrations induced the loss of cell wall integrity in this fungus, resulting in cellular extravasation.

SEM was conducted to explore the effects of the synergistic combinations on the surface structure and morphology of the yeasts studied. Figure 5 shows the results for *C. tropicalis* (Fig. 5A, B, C, and D) and *S. cerevisiae* (Fig. 5E, F, G, and H). Control cells (Fig. 5A, E) and those treated with the synthetic peptide or AmB alone (Fig. 5BC, FG) displayed smooth surfaces without rigidity, furrows, or wrinkles. In contrast, cells treated with synergistic combinations (Fig. 5D, H) showed severe damage, including high levels of roughness, significant changes in morphology, and evident signs of cell lysis, resulting in the loss of cytoplasm. The same effect was observed by Bezerra et al. in 2020, when strains of *C. albicans* and *C. parapsilosis* were treated with combinations of the synthetic peptides Mo-CBP3-PepI + nystatin and Mo-CBP3-PepIII + nystatin.

Their study demonstrated that these synergistic combinations induced morphological changes and resulted in the loss of cytoplasmic components in treated cells.

Lima [18] demonstrated, through scanning electron microscopy analysis, that a synergistic combination of synthetic peptides bioinspired by peptides extracted from *Moringa oleifera* seeds and the antifungal agent nystatin had a remarkable antifungal effect. This synergistic combination increased the anticandidal activity of nystatin, a conventional antifungal, by approximately 50-fold, and induced significant morphological changes in *C. albicans* cells.

Wu [35], in their ultrastructural study, demonstrated that control *C. albicans* cells presented normal cell morphology, with well-defined cell walls, intact nuclei, and numerous membranous organelles. In contrast, cells exposed to PLE, a macrocyclic bis(bibenzyl) with antifungal activity isolated from the liverwort *Marchantia polymorpha* L., showed extensive chromatin condensation into aggregates in the nuclear envelope, a typical sign of apoptosis.

To comprehensively understand how the identified synergistic combinations effectively killed yeast cells studied in this research, ultrastructural analyses were conducted using transmission electron microscopy. As shown in Fig. S1, the data confirmed that the plasma membrane and cell wall remained intact in the control cells and in cells treated with individual concentrations of the synergistic combination of *C. tropicalis* (Fig. S1A, B, and C) and *S. cerevisiae* (Fig. S1E, F, and G).

The 8-fold difference in MIC₅₀ values between *C. tropicalis* (3.12 µM) and *S. cerevisiae* (25 µM) reinforces that susceptibility to antimicrobial peptides is strongly species-dependent. Variations in cell wall architecture and composition, particularly in the organization of β-glucans, chitin, and mannoproteins, can modulate the binding and diffusion of AMPs [36]. Furthermore, differences in plasma membrane lipid composition, including ergosterol content, influence peptide-membrane interactions [37]. The increased vacuolar structures observed in *S. cerevisiae* suggest activation of adaptive responses and possible intracellular sequestration mechanisms, consistent with the recognized robustness of this species in stress pathways and efflux systems.

## Conclusion

The discovery of the synergistic combination of a synthetic peptide and amphotericin B represents a significant advancement in the development of combination therapies for fungal diseases. The synergistic combinations identified in this study not only affected the cell wall integrity of the yeasts tested but also induced the loss of mitochondrial functionality and the degradation of nuclear components. This multifaceted approach highlights the potential of effectively combating fungal infections. The damage observed to the ultrastructure and morphology of the tested cells highlights the importance of studying the synergy between antifungal molecules to improve their activity. In addition, the use of the *S. cerevisiae* model demonstrated the efficacy of these synergistic concentrations in model microorganisms, allowing for the comparison of their activity with that of other more complex microorganisms.

## Supplementary Information

Below is the link to the electronic supplementary material.

Supplementary Material 1

## Data Availability

All data generated or analysed during this study are included in this published article.
