# Supplementary material for: In Vitro Activity and Synergism of Amphotericin B and the Synthetic Bioinspired Peptide γ31-45PvD1++ Against Fungal Study Models Candida tropicalis and Saccharomyces cerevisiae
Source: Curr Microbiol. 2026 Oct 21;83(12):608. doi: 10.1007/s00284-026-05197-2 (PMC13687208; doi:10.1007/s00284-026-05197-2)
Supplement: Supplementary file 1 [file 284_2026_5197_MOESM1_ESM.docx]

***Saccharomyces cerevisiae***

***Candida tropicalis***

**Fig. S1** Ultrastructural changes visualized by transmission electron microscopy in *C. tropicalis* (A-D) and *S. cerevisiae* (E-H) yeast cells. Arrows indicate cytoplasmic disorganization and degradation, and asterisks show leakage of cytoplasmic material. (N) represents the nucleus, (M) the mitochondria, and (V) the vacuoles. Barr = 1 µm.
